# Development and validation of a novel hypoxia-related signature for prognostic and immunogenic evaluation in head and neck squamous cell carcinoma

**DOI:** 10.3389/fonc.2022.943945

**Published:** 2022-11-14

**Authors:** Su-Ran Li, Qi-Wen Man, Bing Liu

**Affiliations:** ^1^ The State Key Laboratory Breeding Base of Basic Science of Stomatology (Hubei-MOST) and Key Laboratory of Oral Biomedicine Ministry of Education, School and Hospital of Stomatology, Wuhan University, Wuhan, China; ^2^ Department of Oral Maxillofacial Head Neck Oncology, School and Hospital of Stomatology, Wuhan University, Wuhan, Hubei, China

**Keywords:** head and neck squamous cell carcinoma, hypoxia, The Cancer Genome Atlas, Gene Expression Omnibus, tumor microenvironment, prognostic signature

## Abstract

Hypoxia plays a critical role in head and neck squamous cell carcinoma (HNSCC) prognosis. However, till now, robust and reliable hypoxia-related prognostic signatures have not been established for an accurate prognostic evaluation in HNSCC patients. This article focused on establishing a risk score model to evaluate the prognosis and guide treatment for HNSCC patients. RNA-seq data and clinical information of 502 HNSCC patients and 44 normal samples were downloaded from The Cancer Genome Atlas (TCGA) database. 433 samples from three Gene Expression Omnibus (GEO) datasets were incorporated as an external validation cohort. In the training cohort, prognostic-related genes were screened and LASSO regression analyses were performed for signature establishment. A scoring system based on SRPX, PGK1, STG1, HS3ST1, CDKN1B, and HK1 showed an excellent prediction capacity for an overall prognosis for HNSCC patients. Patients were divided into high- and low-risk groups, and the survival status of the two groups exhibited a statistically significant difference. Subsequently, gene set enrichment analysis (GSEA) was carried out to explore the underlying mechanisms for the prognosis differences between the high- and low-risk groups. The tumor immune microenvironment was evaluated by CIBERSORT, ESTIMATE, TIDE, and xCell algorithm, etc. Then, we explored the relationships between this prognostic model and the levels of immune checkpoint-related genes. Cox regression analysis and nomogram plot indicated the scoring system was an independent predictor for HNSCC. Moreover, a comparison of predictive capability has been made between the present signature and existing prognostic signatures for HNSCC patients. Finally, we detected the expression levels of proteins encoded by six-HRGs *via* immunohistochemical analysis in tissue microarray. Collectively, a novel integrated signature considering both HRGs and clinicopathological parameters will serve as a prospective candidate for the prognostic evaluation of HNSCC patients.

## Introduction

Head and neck squamous cell carcinoma (HNSCC) ranks sixth among malignant tumors worldwide, with 930,000 new cases in 2020 (1, 2). The incidence of HNSCC keeps rising and is expected to increase by 30% by 2030 (3). HNSCC makes up over 90% of head and neck cancers, and excessive consumption of alcohol and tobacco has been widely recognized as a leading risk factor (1). Over the past decades, although great progress has been made in multidisciplinary therapy, the 5-year overall survival rate of locally advanced HNSCC patients remains around 50% (4–6). Given the growing incidence and poor prognosis of HNSCC, better prognostic tools that allow accurate prediction of tumor progression are an urgent necessity to tailor comprehensive management strategies for HNSCC patients.

In recent decades, a rising number of researches have shown that the tumor microenvironment (TME) was closely related to prognosis in multiple types of cancer, such as HNSCC (7), breast cancer (8), and pancreatic cancer (9). Hypoxic TME is a common feature of solid tumors (10). Hypoxic TME is formed because of excessive metabolism of tumor cells and insufficient oxygen supply compared to under physiological conditions. Hypoxia induces the formation of tumor heterogeneity and promotes the acquisition of more aggressive characteristics (11). Substantial data suggest that hypoxic TME participates in tumorigenesis, angiogenesis, immunosuppression, cancer progression, and treatment resistance due to hypoxic changes and increased tumor heterogeneity (12, 13).

In addition, studies have reported that hypoxia played a crucial role in reprogramming the tumor immune microenvironment (TIME) (14). The hypoxia-induced acidification of TME impairs the proliferation and resultant anti-tumor immune responses of T cells. Collectively, hypoxic TME indicates an unfavorable prognosis (15). Given that hypoxia is actively involved in tumor progression, an HRG-based signature can enrich our knowledge of potential molecular mechanisms and survival evaluation for HNSCC patients.

To date, the TNM staging criterion has been recognized as an authorized system for the evaluation of survival outcomes and a well-acknowledged guideline for the development of treatment regimens (16). However, the TNM evaluation system only considers the macroscopic indicators of the tumor, including size, and lymph node metastasis, coupled with distant metastasis, although intra-tumoral heterogeneity is closely associated with patient prognosis. Additionally, the TNM system could not achieve a prediction prognosis in an accurate and individualized manner. Therefore, a novel prognostic tool is in pressing need for earlier diagnosis and treatment of HNSCC patients (17).

In this study, we constructed a novel and powerful prognostic signature for HNSCC patients by combining hypoxia-related genes (HRGs) expression profiles and patients’ information from The Cancer Genome Atlas (TCGA). Next, we constructed a six-HRG prognostic signature by Cox regression and LASSO regression analyses. The prediction capacity of the HRG-based signature was further validated in GSE65858, GSE41613, and GSE85446 from the Gene Expression Omnibus (GEO) database. Then, we validated that the HRG-based prognostic model was an independent evaluation indicator for HNSCC patients. Furthermore, multiple methods were employed to analyze the relationships between the prognosis and TIME in HNSCC patients. Finally, the protein levels of six HRGs were validated in 127 HNSCC patients and 28 normal cases. In conclusion, we aimed to adopt a systematic and holistic analysis strategy to construct a robust prognostic model based on six HRGs, which could efficiently assess the survival outcomes for HNSCC patients.

## Materials and methods

### Patients and datasets

RNA-seq data of 546 samples, including 502 tumor tissues and 44 normal tissues, and corresponding clinicopathological data were obtained from TCGA database (18). Fragments per kilobase million (FPKM) values were employed in the following analyses. The demographic parameters of the HNSCC patients were given in **Table 1**. RNA-seq data of 433 HNSCC samples (GSE65858, GSE41613, and GSE85446) and the clinical data were obtained from the GEO database (https://www.ncbi.nlm.nih.gov/geo/). In addition, 127 tumor samples were collected from HNSCC patients who underwent surgery at the Department of Oral Maxillofacial Head and Neck Oncology of the Hospital of Stomatology of Wuhan University from 2017.2 to 2018.7. All patients in this study provided informed consent before surgery. The Ethics Committee of School & Hospital of Stomatology, Wuhan University approved this study (IRB-ID: 2021B56). The samples from TCGA included 44 normal tissues and 502 tumor tissues in this study. The RNA-seq data and clinical information were processed using R (version 4.0.4). Protein-coding genes were annotated and classified using the Ensemble human genome browser GRCh38.p13 (http://asia.ensembl.org/index.html) (19). patients with incomplete prognostic information were excluded from our study.

**Table 1:** Demographic data from TCGA patients.

			High Risk		Low Risk	
			N	%	N	%
**Age (Years)**			260		239	
	≥60	279	157	60.4	122	51.0
	<60	220	103	39.6	117	49.0
**Gender**						
	Male	366	180	69.2	186	77.8
	Female	133	80	30.8	53	22.2
**Grade**						
	1	61	30	11.5	31	13.0
	2	298	167	64.2	131	54.8
	3	119	56	21.6	63	26.4
	4	2	0	0.0	2	0.8
	X	16	6	2.3	10	4.2
	Unknown	3	1	0.4	2	0.8
**Stage**						
	1	25	11	4.2	14	5.9
	2	79	42	16.2	37	15.5
	3	89	39	15.0	50	20.9
	4A	287	159	61.1	128	53.6
	4B	13	7	2.7	6	2.5
	4C	3	1	0.4	2	0.8
	Unknown	3	1	0.4	2	0.8
**T**						
	0	1	0	0.0	1	0.4
	1	47	16	6.2	31	13.0
	2	148	72	27.7	76	31.8
	3	114	62	23.8	52	21.8
	4	184	108	41.5	76	31.8
	X	4	1	0.4	3	1.2
	Unknown	1	1	0.4	0	0.0
**M**						
	0	484	250	96.2	234	98.0
	1	4	3	1.1	1	0.4
	X	11	7	2.7	4	1.6
**N**						
	0	212	118	45.4	94	39.3
	1	75	30	11.5	45	18.8
	2	197	104	40.0	93	38.9
	3	9	5	1.9	4	1.7
	X	5	2	0.8	3	1.3
	Unknown	1	1	0.4	0	0.0
**Race**						
	AI/AN	2	0	0.0	2	0.8
	Asian	10	6	2.3	4	1.7
	Black	47	22	8.5	25	10.5
	White	426	224	86.1	202	84.5
	Unknown	14	8	3.1	6	2.5
**HPV**						
	Unknown	397	216	83.1	181	75.7
	Negative	72	39	15.0	33	13.8
	Positive	30	5	1.9	25	10.5
						
**Smoke**						
	Yes	378	201	77.3	177	74.1
	No	111	53	20.4	58	24.3
	NA	10	6	2.3	4	1.6
**Sample Type**						
	Primary	499	260	100.0	239	100.0
	Metastatic	0	0	0.0	0	0.0

AI/AN, American Indian or Alaska Native.

### Differentially expressed hub HRGs

The differential expression of HRGs between HNSCC patients and the control group was determined by the “limma” package, with the cut-off value of |log_2_ fold change| > 1 and false discovery rate < 0.05. Gene Ontology (GO) and gene set enrichment analysis (GSEA) enrichment analyses were performed using the “clusterprofiler” package to explore the underlying molecular mechanisms of these hub HRGs. The “ggplot2” and “GOplot” packages in R software were employed to visualize GO and GSEA analyses results.

### Establishment of the prognostic HRG-based signature

Univariate Cox regression analysis was performed to screen prognosis-related HRGs using the “survival” package. *P*-value < 0.001 was considered an enormous significant difference. The samples from TCGA were divided randomly into two cohorts, the training cohort (n=251) and the validation cohort (n=248). Univariate Cox analysis was utilized to identify prognosis-associated HRGs. LASSO Cox regression analysis was performed using the “glmnet” tool in the R package to construct a prognostic signature using survival-related HRGs in the training cohort. Risk scores were calculated using the obtained coefficients and corresponding expression levels (risk score = Σ relative expression (mRNAs) * coefficient). Subsequently, HNSCC patients were divided into high- and low-risk groups based on the median risk score. By using the R package “survival”, Kaplan–Meier survival analysis and a log-rank test were performed to evaluate the association between each HRG and patient survival.

### Evaluation of the prognostic value of the HRG-based signature

Patients were assigned to the high- and low-risk groups based on the median score for the six-HRG signature. Next, we conducted Kaplan–Meier survival analysis to assess the association between HRG-based prognostic signature and patient survival in the validation cohort. The three GEO datasets were employed as an external validation cohort to assess the predictive value of the six-HRG signature. Furthermore, univariable and multivariable Cox regression analyses with comprehensive clinicopathological information were utilized to test whether the present signature was an independent factor.

### Evaluation of infiltrating immune cells, immune score, and stromal score

TIMER, MCP-count, EPIC, quanTIseq, and xCell algorithms were used to analyze the relative abundance of infiltrating immune cells in each HNSCC patient. Next, a Wilcoxon test was employed to assess the differences in the degree of immune cell infiltration between the high- and low-risk groups, and visualized by the “fmsb” package. ESTIMATE algorithm was applied for the evaluation of the cell abundance within TME and then calculated the Immune Score, Stromal Score, and ESTIMATE Score based on the gene expression of high- and low-risk groups (20).

### Evaluation of immunotherapy

Moreover, for further analysis of the relationship between the TME and immunotherapy efficiency, we selected a panel of critical immune checkpoint molecules to explore the expression of these candidates in high- and low-risk groups and then evaluate potential treatment responses. The responses to immune checkpoint inhibitors (ICI) were evaluated by the Cancer Immunome Database. The differences between the two groups were discovered using the Wilcoxon test.

### Genetic alteration among subgroups

Mutation data of HNSCC patients was obtained from TCGA database. The top 20 genes with the highest mutation frequency were identified. Oncoplots were sketched in the high- and low-risk groups by “maftools” package.

### Validation of independence and forecast efficiency of prognostic signature

To determine whether the HRG-based prognostic signature could be an independent tool for HNSCC patients, univariate and multivariate Cox regression analyses were employed, with age, gender, stage, T stage, N stage, M stage, and risk score as variates. Then, we developed a nomogram based on risk score and clinical indicators to evaluate the survival probability of 1-, 3-, and 5-year for HNSCC patients using the “rms,” “foreign,” and “survival” packages.

### Immunohistochemical (IHC) staining

We employed an HNSCC tissue microarray, which contains 28 oral mucosa tissues, 13 atypical hyperplasia tissues, and 127 HNSCC tissues to further confirm the relationship between genes, prognosis, and clinical features. Antibodies of SRPX (A1217, Wuhan, Abclonal), PGK1 (17811-1-AP, Wuhan, Proteintech), STC1 (A6755, Wuhan, Abclonal), HS3ST1 (14358-1-AP, Wuhan, Proteintech), CDKN1B (25614-1-AP, Wuhan, Proteintech), HK1 (19662-1-AP, Wuhan, Proteintech) were used for IHC staining. IHC staining was carried out as we previously described (21). In brief, the 4-μm sections were dewaxed, rehydrated, antigen-retrieved, and blocked. Subsequently, the sections were incubated with the primary antibody at 4˚C overnight. Then corresponding second antibodies were incubated. Subsequently, DAB staining and Hematoxylin staining were performed. Finally, the sections were observed and scanned with a digital section scanner (3DHISTECH, Hungary) and analyzed by ImageScope software (Leica). The histoscore of each slide was calculated according to the formula: (percentage of strong positive cells) × 3 + (percentage of positive cells) × 2 + (percentage of weak positive cells) × 1)/total cell number.

### Statistical analysis

All data were analyzed by R software (version 4.0.4). *P* < 0.05 was determined as statistically significant. The independent Student’s t-test for continuous data and the χ^2^ test for categorical data were employed for pairwise comparisons between the two groups. The log-rank test was utilized to compare two groups in Kaplan-Meier survival curves. The Wilcoxon test was used to compare the abundance difference of tumor-infiltrating immune cells in both groups.

## Results

### Identification of hypoxia-related genes

**Figure 1:**
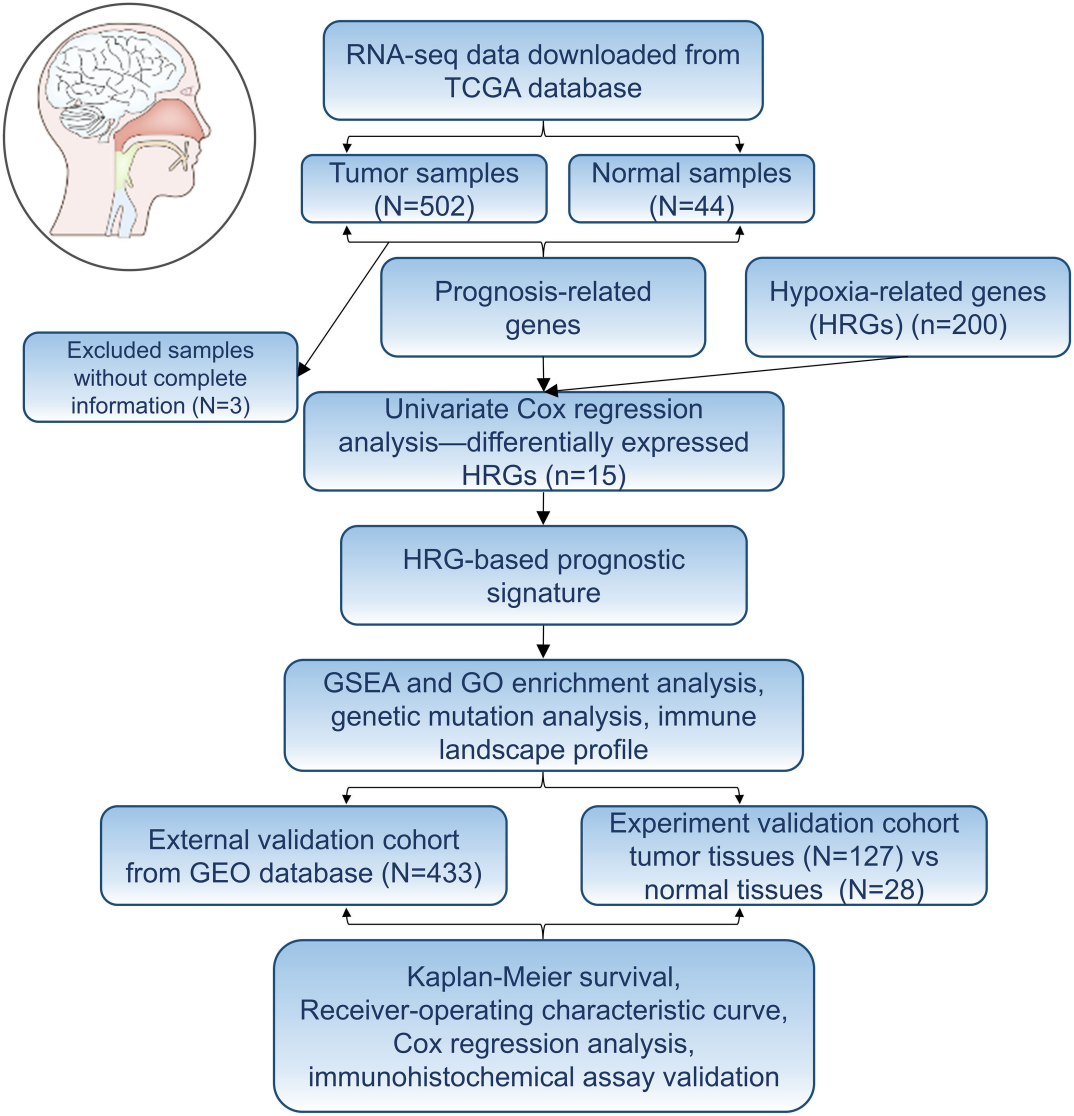
Flowchart of establishing a prognostic signature of head and neck squamous cell carcinoma in this study.

The whole research process was presented in **Figure 1**. 56, 753 genes were identified by analyzing the RNA-Seq data from TCGA comprising 546 cases, including 502 cancer cases and 44 non-cancer cases. A total of 200 genes were screened as HRGs. Then, Kaplan–Meier analysis was performed to evaluate the relationship between single HRG and patient survival. Results showed that prognostic evaluation for HNSCC based on a single gene may be unstable, thus an effective prognostic signature based on multiple genes was necessary.

### Establishment of the predictive six-HRG signature

**Figure 2:**
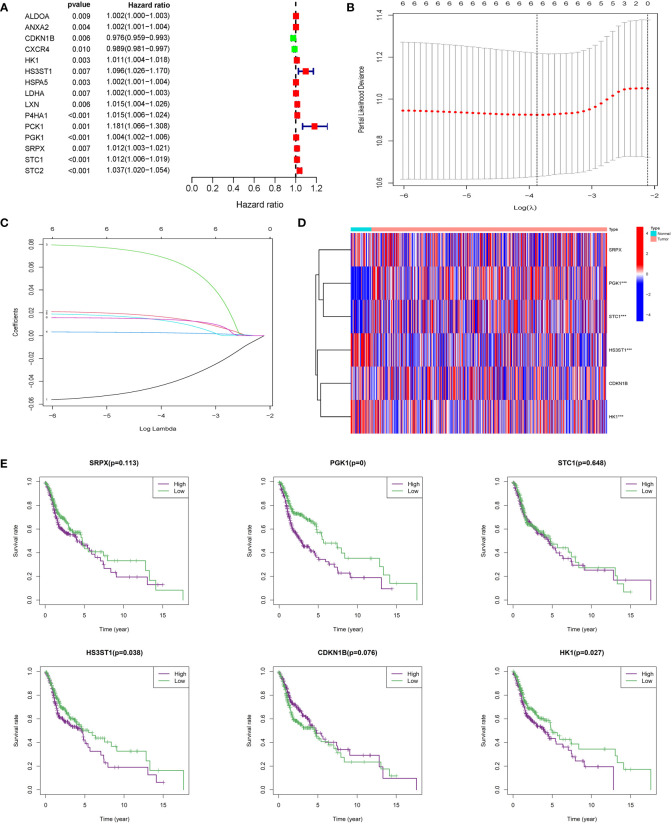
Construction of integrated risk score based on HRGs. **(A)** ALDOA, ANXA2, CDKN1B, CXCR4, HK1, HS3ST1, HSPAS, LDHA, LXN, P4HA1, PCK1, PGK1, SRPX, STC1, and STC2 were significantly correlated with clinical prognosis in univariate Cox regression model. **(B, C)** The LASSO Cox analysis identified six genes associated with prognosis. **(D)** SRPX, PGK1, STC1, HS3ST1, CDKN1B, and HK1 were differentially expressed between normal tissues and HNSCC tissues. **(E)** Kaplan–Meier survival curves were plotted for each of the six genes to predict patient outcomes.

200 HRGs in HNSCC patients were screened from the Molecular Signatures Database (MSigDB version 6.0). 15 differentially expressed HRGs were considered to be significantly related to the prognosis of HNSCC patients. CDKN1B (*HR*: 0.976, 95% *CI*: 0.959−0.993, *P* = 0.006) and CXCR4 were protective factors, and the other 13 HRGs were risk factors (**Figure 2A**), such as HK1 (*HR*: 1.011, 95% *CI*: 1.004−1.018, *P* = 0.003) and HS3ST1 (*HR*: 1.096, 95% *CI*: 1.026−1.170, *P* = 0.007). LASSO regression analysis was conducted to construct an excellent HRG-based prognostic model for HNSCC patients (**Figures 2B, C** **)**. Then, six HRGs (SRPX, PGK1, STC1, HS3ST1, CDKN1B, and HK1) were selected (**Figure 2D**). Among these selected HRGs, CDKN1B was down-regulated, and other genes were the opposite in HNSCC tissues compared to their counterparts in TCGA database. As expected, single gene-based model showed poor efficiency for prognostic evaluation, such as SRPX (*P* = 0.113), STC1 (*P* = 0.648), CDKN1B (*P* = 0.076) (**Figure 2E**). In contrast, the prognostic signature based on six HRGs exhibited more robust evaluation efficiency.

### Validation of the prognostic signature

**Figure 3:**
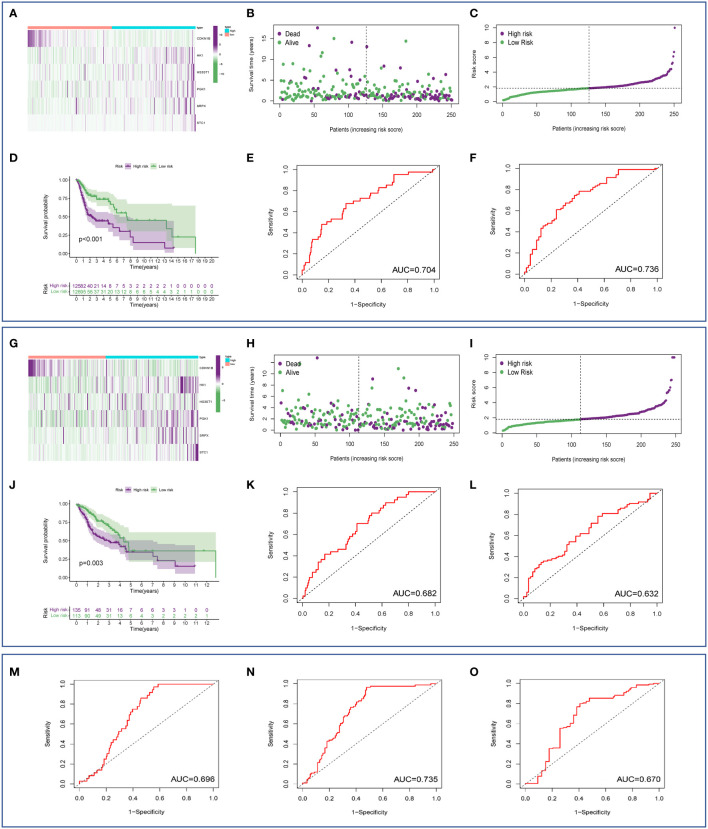
Estimation of the prognostic model based on HRGs. **(A)** Heatmap of 6 HRGs in the training set. **(B)** Scatterplot of patient survival in the training cohort. **(C)** Survival rate was significantly associated with risk score in the training cohort. **(D)** Kaplan-Meier survival curves of the high- and low-risk groups in the training cohort. **(E, F)** ROC curves based on the risk score model in the training cohort. **(G)** Heatmap of 6 HRGs in the validation cohort. **(H)** Scatterplot of patient survival in the validation cohort. **(I)** Survival rate was significantly associated with risk score in the validation cohort. **(J)** Kaplan-Meier survival curves of the high-risk group and the low-risk group in the validation cohort. **(K, L)** ROC curve based on the risk score model in the validation cohort. **(M–O)** External validation (GEO database) of an HRG-based prognostic signature.

HNSCC patients from TCGA were divided into a high-risk group (n=260) and a low-risk group (n=239) based on the median risk score. In the training cohort, the heatmap of 6 HRGs showed a significantly higher expression of HK1, HS3ST1, PGK1, SRPX, and STC1 in the high-risk group (**Figure 3A**). A scatterplot of patient survival showed a significantly decreased survival time as the risk score increased (**Figure 3B**). Survival rate was significantly related to risk score in the training cohort (**Figure 3C**). Kaplan-Meier survival analysis of the high-risk group and the low-risk group was performed in the training cohort (*P* < 0.001) (**Figure 3D**). 1-year and 3-year survival ROC curves in the training cohort were presented, and AUC values were 0.704 and 0.736, respectively (**Figures 3E, F**). The validation cohort was applied to verify the accuracy of prognostic prediction. Heatmap of 6 HRGs (**Figure 3G**), scatterplot of patient survival (**Figure 3H**), survival rate analysis (**Figure 3I**), Kaplan-Meier survival curves of the high- and low-risk group (*P* = 0.003) (**Figure 3J**), as well as 1-year and 3-year survival ROC curves (**Figures 3K, L**). Additionally, to verify the evaluation capacity of the model, three external validation cohorts (GSE65858, GSE41613, GSE85446) were integrated as a whole for 1-, 3-, and 5-year survival analyses. ROC curves were plotted to evaluate the predictive value of the prognostic signature. Expectedly, the AUC values of the 1-, 3-, and 5-year ROC curves were 0.696, 0.735, and 0.670, respectively (**Figures 3M–O**), which supported our results that this model had good accuracy for prognostic evaluation of HNSCC patients.

**Figure 4:**
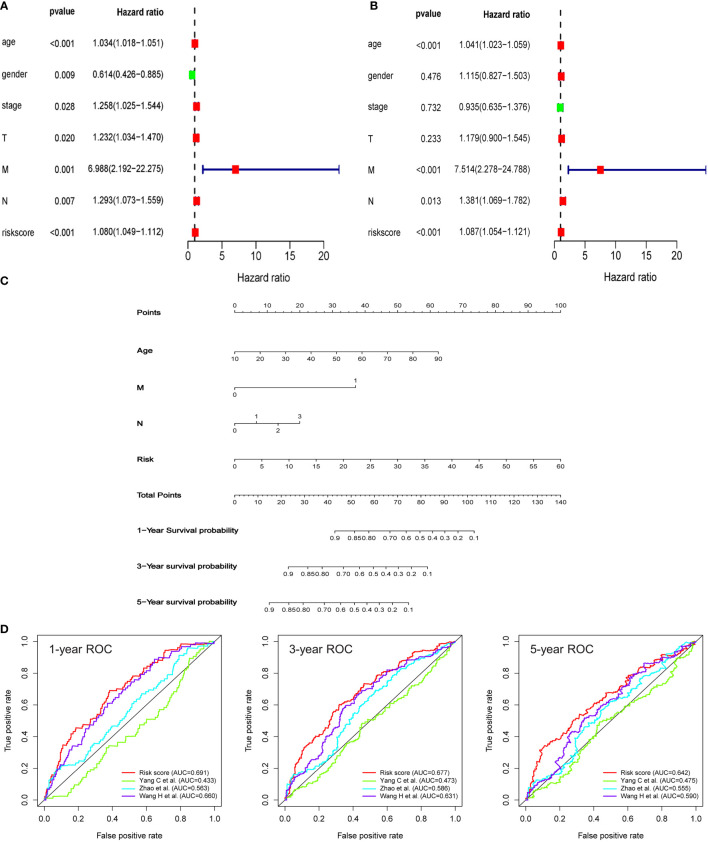
An HRG-based signature as an independent prognostic model. **(A, B)** The univariate and multivariate Cox regression models indicated that the risk score was an independent prognostic predictor for overall survival. **(C)** A nomogram to predict survival probability at 1-, 3‐, and 5‐year after surgery for HNSCC patients based on the results deriving from the entire set. **(D)** ROC curves for different prognostic signatures. The AUC values for the present signature of 1-year, 3-year, and 5-year were 0.691, 0.677, and 0.642, respectively, and these values were significantly higher than another three existing models.

To explore whether the signature can serve as a clinically independent prognostic factor for HNSCC patients, we performed univariate and multivariate Cox regression analyses. We found that age (*HR*: 1.034, 95% *CI*: 1.018-1.051, *P* < 0.001), gender (*HR*: 0.614, 95% *CI*: 0.426-0.885, *P* = 0.009), clinical stage (*HR*: 1.258, 95% *CI*: 1.025-1.544, *P* = 0.028), T stage (*HR*: 1.232, 95% *CI*: 1.034-1.470, *P* = 0.020), M stage (*HR*: 6.988, 95% *CI*: 2.192-22.275, *P* = 0.001), N stage (*HR*: 1.293, 95% *CI*: 1.073-1.559, *P* = 0.007), and riskscore (*HR*: 1.080, 95% *CI*: 1.049-1.112, *P* < 0.001) demonstrated excellent prognostic value in univariate Cox regression analysis. Of these parameters, gender was the only protective factor (**Figure 4A** **)**. By contrast, in multivariate Cox regression analysis, age (*HR*: 1.041, 95% *CI*: 1.023-1.059, *P* < 0.001), M stage (*HR*: 7.514, 95% *CI*: 2.278-24.788, *P* < 0.001), N stage (*HR*: 1.381, 95% *CI*: 1.069-1.782, *P* = 0.013), and risk score (*HR*: 1.087, 95% *CI*: 1.054-1.121, *P* < 0.001) could serve as independent predictors for patients with HNSCC (**Figure 4B**). Then, we incorporated these indicators with significant prediction values, including age (*P* < 0.001), N stage (*P* < 0.001), M stage (*P* = 0.013), and risk score (*P* < 0.001), to establish a nomogram model based on the entire cohort for prognostic evaluation of HNSCC patients by combining this model with other clinicopathological parameters, including age and tumor stage. According to the total number of points in the nomogram, we could offer an individualized and accurate risk evaluation for HNSCC patients (**Figure 4C**). Results confirmed that this signature could serve as a powerful and reliable tool for the prognostic assessment of HNSCC patients. Finally, a comparative analysis has been made to explore the differences in prognostic evaluation between the present model and existing prognostic models. Results demonstrated that AUC values of 1-year (AUC=0.691), 3-year (AUC=0.677), and 5-year (AUC=0.642) were higher than that of study by Yang C et al. (AUC=0.433, 0.473, 0.475) (22), Zhao et al. (AUC=0.563, 0.586, 0.555) (23), and Wang H et al. (AUC=0.660, 0.631, 0.590) (24) (**Figure 4D**).

### Pathway enrichment analysis

**Figure 5:**
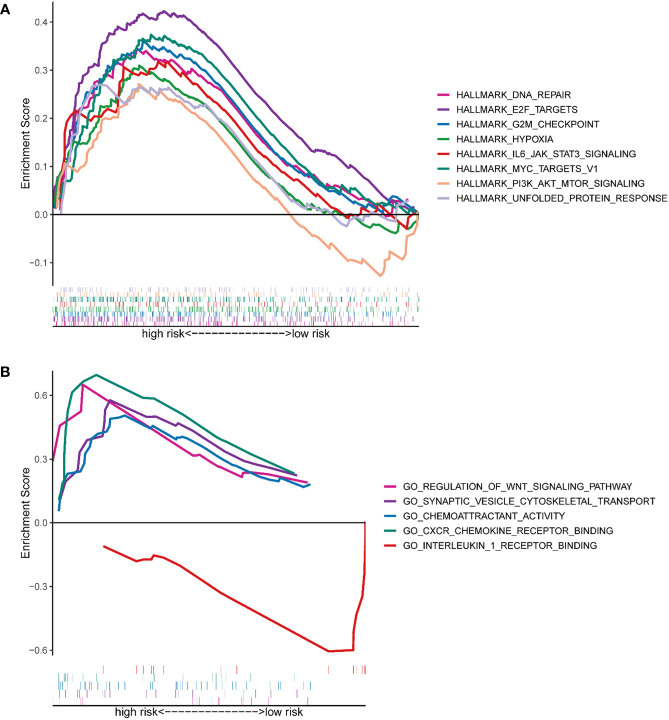
Functional prediction of the HRG-based signature *via* GSEA. **(A)** The hallmark gene set was enriched between the high- and low-risk groups. **(B)** The GO pathway set was enriched between the high- and low-risk groups.

GSEA analysis was conducted to uncover candidate pathways involved in hypoxia. Results of the GSEA analysis identified several pathways enriched in the high-risk group, including DNA repair signaling pathways, E2F targets, G2M checkpoint, hypoxia signaling, PI3K-AKT-mTOR signaling, etc. **(** **Figure 5A** **)**. Pathways enriched in the high-risk group were related to cell proliferation, such as DNA repair signaling pathways, E2F targets, G2M checkpoint, and PI3K-AKT-mTOR signaling. For example, members of the E2F family are well-established candidates for the regulation of DNA damage-response and checkpoint controls. Direct binding between E2F and tumor suppressive molecules as well as other genes has been found, which leads to the loss of gene stability and then the formation of cancers (25). GO analysis highlighted that HRGs with a statistical difference were enriched in the regulation of the WNT signaling pathway, synaptic cytoskeletal transport, chemoattractant activity, CXCR chemokine receptor binding, and interleukin 1 receptor binding (**Figure 5B** **)**. It was reported that hypoxia, in lung cancer, activated the WNT signaling pathway by increasing the stability of β-catenin and translocating it into the nuclear to promote the expression of downstream target genes in a HIF-2α-dependent manner. And subsequently, a series of essential events, including cell migration, invasion as well as colony formation were enhanced in the hypoxia microenvironment (26). Chronic hypoxia elevated the expression of CXCR1 and CXCR2 in prostate cancer cells and then CXCRs promoted the secretion of IL-8. Activation of IL-8 signaling was demonstrated to promote tumor growth and progression, and on the other hand, blocking the IL-8 signaling pathway will improve treatment responsiveness (27). Moreover, hypoxia was demonstrated to facilitate breast carcinoma invasion by inhibiting glycogen synthase kinase 3β (GSK-3β) activity, increasing microtubule stability, and regulating the microtubule-dependent trafficking of Rab11-containing vesicles, and finally Rab11 targeted to the integrin α6β4 to promote carcinoma cells migration (28). In short, pathway enrichment analysis showed that hypoxia was closely related to the growth and invasion of tumors, which represented a poor prognosis.

### The correlation between the prognostic signature and genetic mutations

**Figure 6:**
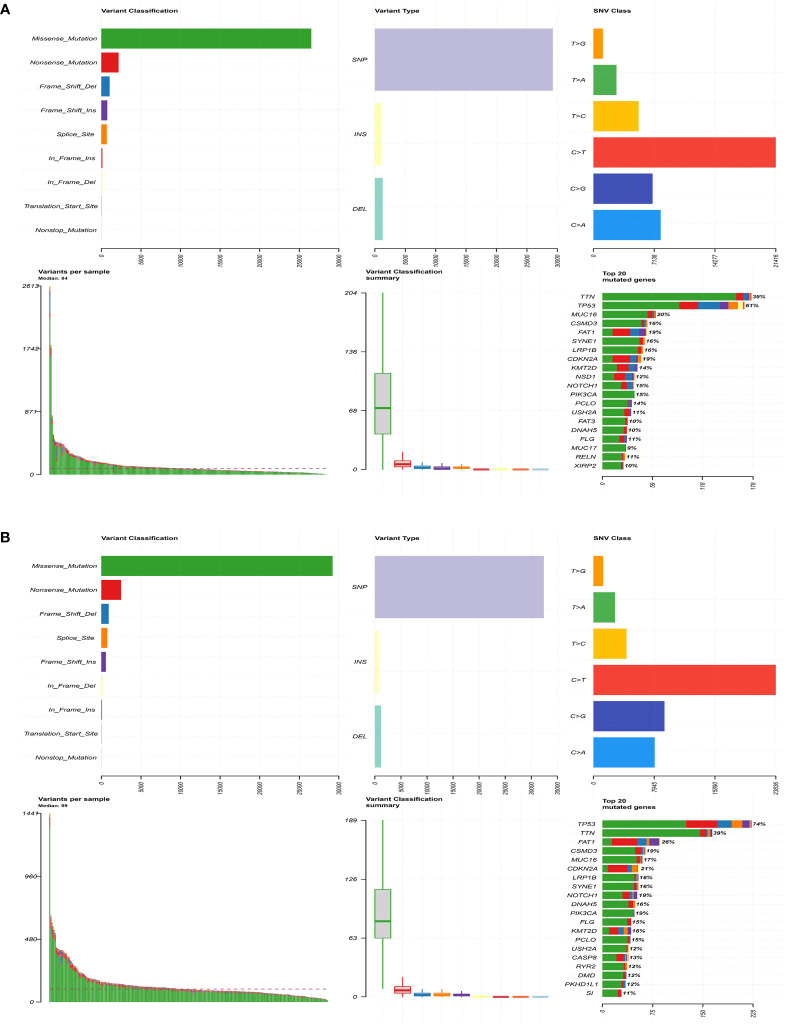
Comparison of genetic mutations in the high- and low-risk group. **(A)** The top 20 most frequently mutated genes in the low-risk group. **(B)** The top 20 most frequently mutated genes in the high-risk group.

Next, gene mutation analysis was performed to gain in-depth knowledge of the immunological characteristics of both groups. We found a higher mutation frequency in the high-risk group (*P* = 0.002). The missense mutation was the most common among all mutation types, followed by the nonsense mutation. We then presented the top 20 genes with the highest mutation frequency in both groups. The mutation rates of TP53, TTN, FAT1, CSMD3, MUC16, CDKN2A, and LRP1B were over 15% in both groups, with TTN and TP53 genes being the highest in both groups (**Figure 6**). Furthermore, the mutation frequency of PIK3CA and FLG showed statistically significant differences between the high- and low-risk groups (**Table 2**). PIK3CA is the most commonly mutated oncogene in HNSCC and encodes a catalytic subunit of phosphatidylinositol 3-kinase (PI3K). Overexpression of PI3K can cause activation of the Akt/mTOR signaling pathway (29). Hyperactivation of PI3K/Akt/mTOR signaling is often related to therapy resistance (30). In our research, PIK3CA showed a higher mutation rate in the high-risk group, indicating a poorer treatment response.

**Table 2:** Differences in mutated genes between high and low risk groups.

Gene symbol	High-risk group		Low-risk group		P Value	
	Count	%	Count	%		
TP53	163	70	163	65	0.228937512		
TTN	91	39	95	38	0.710814205		
FAT1	56	24	53	21	0.418859743		
CDKN2A	54	23	45	18	0.146332653		
MUC16	42	18	48	19	0.689839556		
CSMD3	42	18	45	18	0.800663644		
PIK3CA	49	21	35	14	0.038182444		*
NOTCH1	47	20	35	14	0.065342009		
SYNE1	44	19	45	18	0.711679777		
LRP1B	42	18	33	13	0.132656887		
KMT2D	37	16	35	14	0.516526375		
PCLO	33	14	38	15	0.693726627		
NSD1	30	13	23	9	0.183106551		
DNAH5	30	13	35	14	0.669842237		
USH2A	23	10	35	14	0.160947054		
FLG	37	16	25	10	0.049515551		*
CASP8	23	10	28	11	0.600298919		
PKHD1L1	28	12	20	8	0.130834019		
RYR2	26	11	25	10	0.61897503		
XIRP2	28	12	18	7	0.066587042		

### Tumor immune landscape between low- and high-risk HNSCC patients

**Figure 7:**
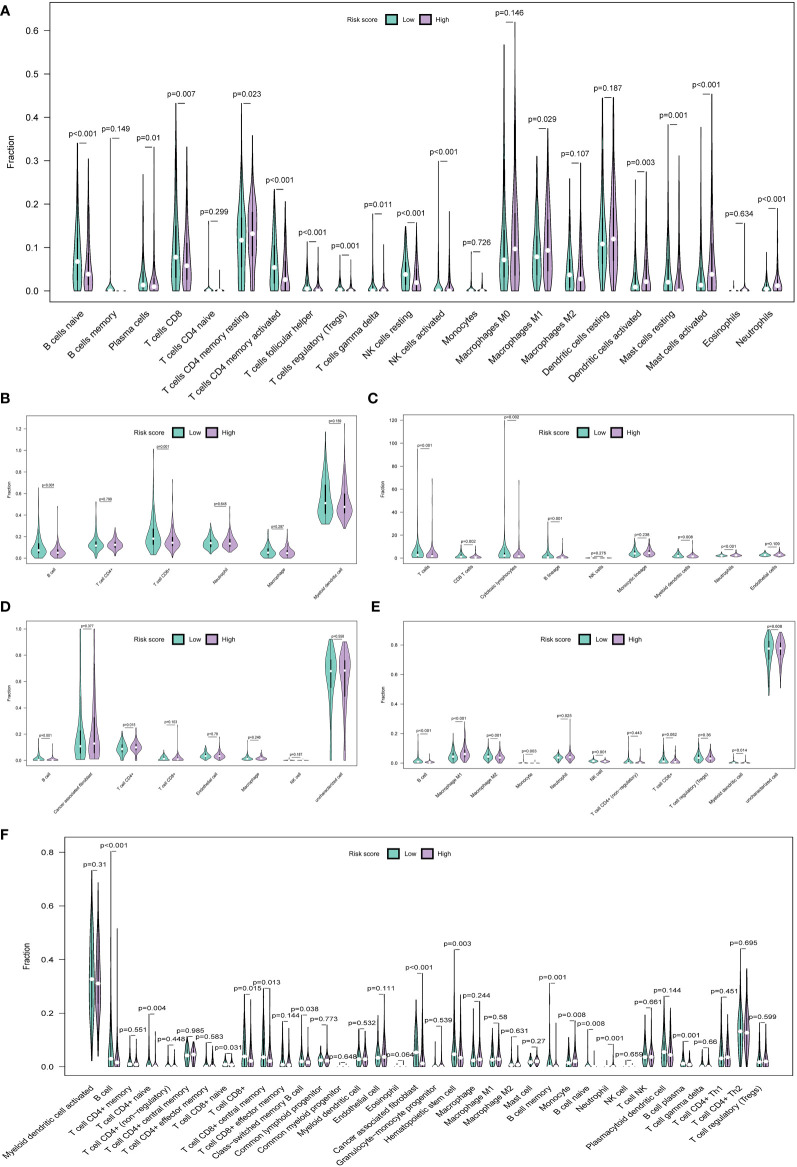
The immune landscape of the TME in HNSCC and the differences between the high- and low-risk groups. Multiple violin plots showed the abundance of infiltration immune cells in the high- and low-risk groups in the CIBERSORT algorithm **(A)**, TIMER algorithm **(B)**, MCP-count algorithm **(C)**, EPIC algorithm **(D)**, quanTIseq algorithm **(E)**, and xCell algorithm **(F)**.

Multiple algorithms were used to` estimate the TIME. We used the CIBERSORT algorithm to analyze the abundance of 22 types of infiltrating immune cells in each HNSCC patient. The abundance of 15 infiltrative immune cell types showed a significant difference between the two groups. Specifically, the infiltration levels of B cells naive, plasma cells, T cells CD8, T cells CD4 memory activated, T cells follicular helper, T cells regulatory, NK cells resting, Mast cells resting, etc. were significantly lower in the high-risk group, whereas T cells CD4 memory resting, macrophages M1, dendritic cells activated, mast cells activated, and neutrophils were the opposite (**Figure 7A**). TIMER provides an open platform to assess the abundance of infiltrating immune cells in TME (http://cistrome.org/TIMER). Results showed that the abundance of B cells (*P* < 0.001) and CD8^+^ T cells (*P* = 0.001) were lower in the high-risk group (**Figure 7B**). Additionally, other algorithms were also used to analyze the TIME, such as MCP-count (**Figure 7C**), EPIC (**Figure 7D**), and quanTIseq (**Figure 7E**). Additionally, xCell analysis showed that 13 out of 36 types of immune cells were significantly different. B cells (*P* < 0.001), T cells CD4^+^ naïve (*P* = 0.004), T cells CD8^+^ naïve (*P* = 0.031), T cells CD8^+^ (*P* = 0.015), cancer-associated fibroblasts (*P* < 0.001), neutrophils (*P* = 0.001), and B cells plasma (*P* = 0.001) were decreased in the high-risk group (**Figure 7F**). The abundance of B cells exhibited a consistent trend between the two groups (*P* < 0.05) in all the above algorithms. These results further supported this six-HRG-based prognostic signature as an excellent tool for profiling immune cell infiltration in HNSCC patients.

**Figure 8:**
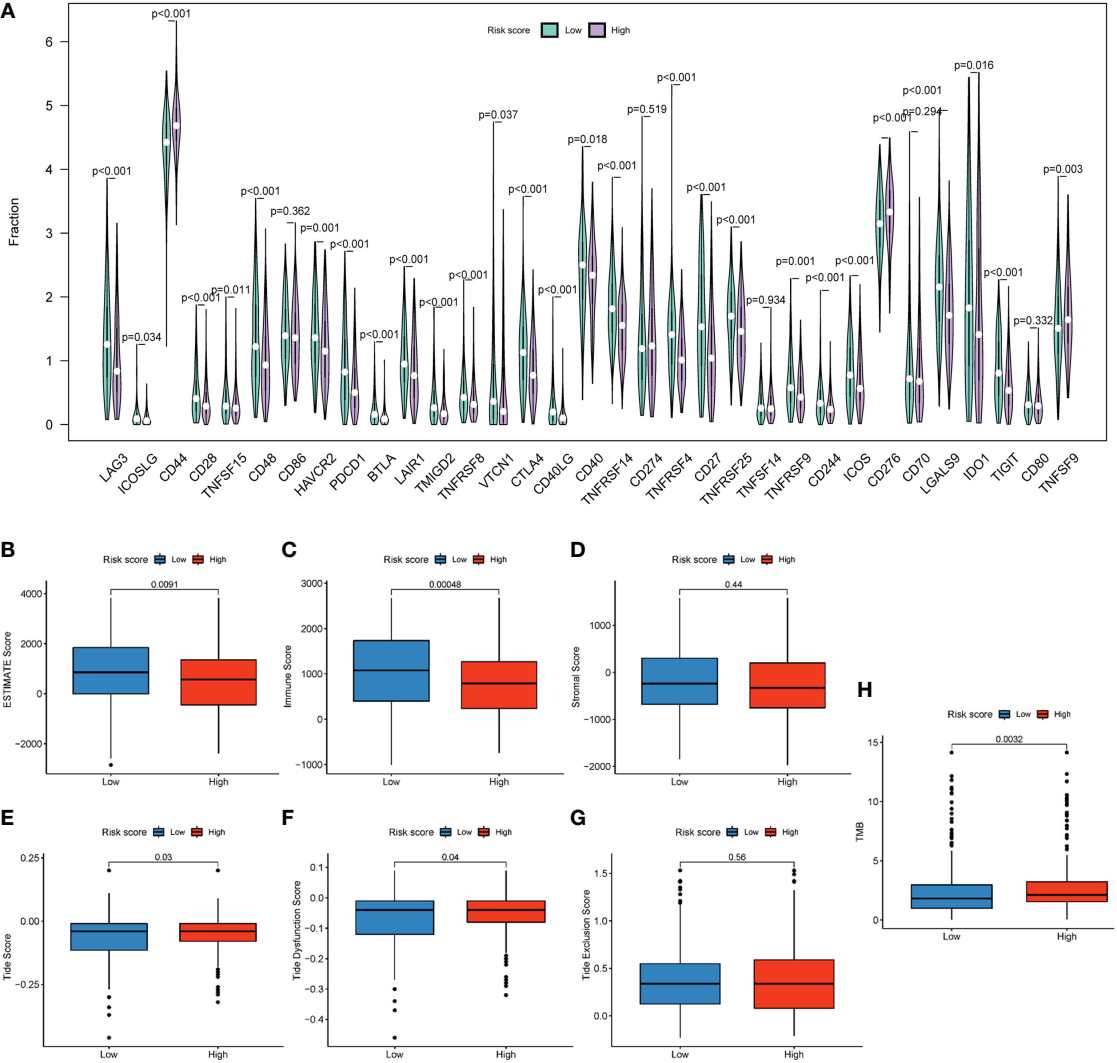
An HRG-based prognostic signature for the evaluation of immune checkpoint therapy. **(A)** The expression of 37 immune checkpoint genes. **(B)** Boxplot showed differences between different groups in the ESTIMATE score. **(C)** Boxplot showed differences between different groups in the stromal score. **(D)** Boxplot showed differences between different groups in the immune score. Boxplot showed differences of different groups in the Tide score **(E)**, TIDE Dysfunction score **(F)**, and TIDE Exclusion score **(G)**. **(H)** Boxplot showed differences in TMB score.

Immune checkpoint genes (ICGs), such as CTLA-4 and PD-1/PD-L1, have been considered potential targets for ICI therapy (31). In our study, we explored the expression of 33 types of ICGs (**Figure 8A**). The majority of ICGs exhibited lower expression in patients in the high-risk group, such as LAG3, CD28, CTLA-4, etc. Downregulated expression of ICGs implied fewer lymphocyte infiltration and poor prognosis (31). Moreover, ESTIMATE serves as a method for the evaluation of the abundance of stromal and immune cells in tumor tissues (32). In our study, the risk score was negatively correlated with ESTIMATE scores (*P* = 0.009) (**Figure 8B**) and the immune score (*P* = 0.00048) (**Figure 8C**), while the stromal score showed no significant difference (*P* = 0.44) (**Figure 8D**), suggesting that the lower the degree of immune cell infiltration, the higher the risk score. In other words, the immune score and ESTIMATE score can be powerful indicators for prognostic evaluation. Interestingly, the TIDE score can be a good evaluation of the efficiency of ICI therapy (anti-PD1 and anti-CTLA4). A higher TIDE score declares a greater chance of immune escape and a lower ICI therapy reactivity (33). In this study, the Tide score and Tide Dysfunction score were elevated in the high-risk group (**Figures 8E, F**), with the Tide Exclusion score having no significant difference (**Figure 8G**). The tumor mutation burden (TMB) can be a helpful tool in predicting responses to ICI therapy (34). Patients in the high-risk group showed a higher TMB score (*P* = 0.0032) (**Figure 8H**). These results demonstrated that patients in the high-risk group may show poor responses to ICI therapy.

### External validation of the expression of the six HRGs in HNSCC samples

**Figure 9:**
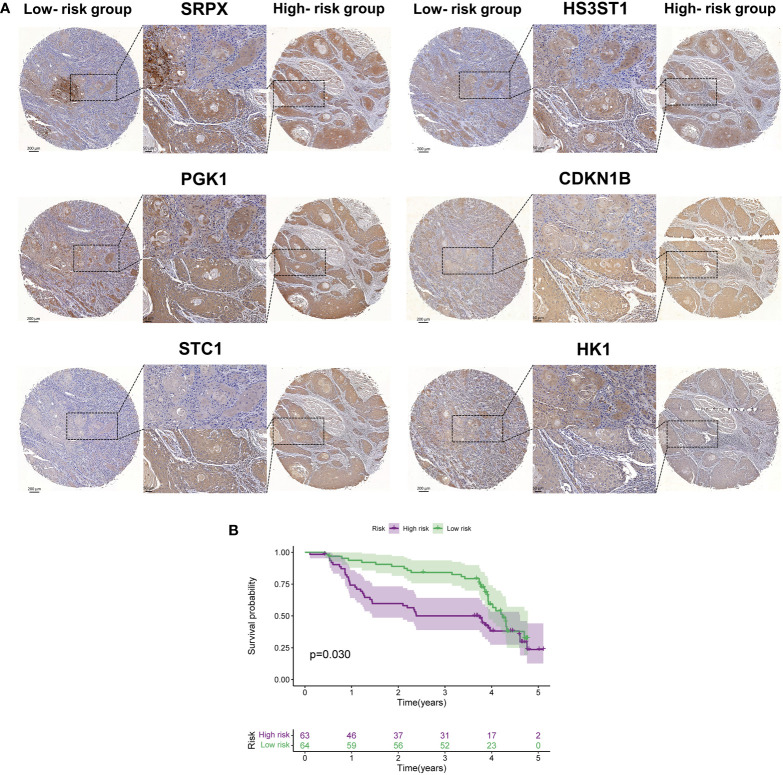
The external validation of proteins encoded by six hub HRGs. **(A)** The expression of six HRGs in the high- and low-risk groups in HNSCC patients. **(B)** Kaplan-Meier survival curves of HNSCC patients in the high- and low-risk groups.

Next, we utilized IHC staining to detect the expression of the proteins encoded by the six HRGs in HNSCC samples. According to the formula (risk score = Σ relative expression (mRNAs) * coefficient), we calculated the risk score for each specimen, and the high- and low-risk groups were divided based on the median. SRPX, PGK1, STC1, HS3ST1, HK1, and CDKN1B were chosen as candidates, and their expressions in both groups were presented. Compared with tissues of the low-risk group, the relative protein levels of SRPX, PGK1, STC1, HS3ST1, and HK1 exhibited significantly higher expression in the other group, which was consistent with the results found in TCGA database. Of note, CDKN1B, a tumor suppressor gene, was overexpressed in tissues of the high-risk group in the clinical validation cohort but a lower expression of CHKN1B was found in the high-risk group in TCGA. As indicated in the Human Protein Atlas (HPA) database (www.proteinatlas.org), CDKN1B is mainly located in the nucleus and involved in the cellular transition toward a proliferative state. PGK1, HS3ST1, and HK1 are intracellular enzymes located in various organelles, such as the Golgi apparatus and mitochondria. SRPX and STC1 are secreted into extracellular space. These hypoxia-related indicators are participated in cellular metabolism and activated in a hypoxic microenvironment. Thus, the expression of the above molecules was modestly increased in regions with higher cell density (**Figure 9A**). Next, we plotted Kaplan-Meier survival curves of the high- and low-risk groups in our patients (*P* = 0.03) (**Figure 9B**), which verified the results of TCGA that patients with higher risk scores suffered an unfavorable prognosis.

## Discussion

Although great advances have been witnessed in surgical therapy, chemotherapy, and radiation therapy, the overall prognosis of HNSCC patients has not been significantly improved due to a lack of sensitive and specific diagnosis biomarkers, and common therapy resistance (35). Therefore, it is necessary to define an effective assessment signature for accurate prediction of the tumor progression and survival condition, coupled with treatment responses of HNSCC patients.

Hypoxia, a hallmark of solid tumors, plays wide-range effects on angiogenesis, metabolism, proliferation, metastasis, and cell differentiation (36). So far, a few HRG-based prognostic signatures have been constructed in various types of cancer, such as breast cancer (37), bladder cancer (38), hepatocellular carcinoma (39), etc. However, robust and reliable prediction models for HNSCC are insufficient. Therefore, a more effective prognostic model for HNSCC patients is urgently needed.

Herein, our study made a comprehensive integration of RNA-seq data from multiple datasets to construct a prognostic signature based on 6 HRGs (SRPX, PGK1, STG1, HS3ST1, CDKN1B, and HK1) in HNSCC. In comparison with a signature based on a single gene, our prognostic signature demonstrated higher stability in predicting tumor pathological characteristics (tumor stage and grade in both clinical and pathological levels) as well as prognosis. Then, an independence test of this prognostic signature was performed. GSEA analysis showed that hub HRGs were largely associated with tumor growth and invasion. Specifically, the TIME of the high- and low-risk groups divided by HRG-based signature was defined by multiple algorithms. For example, we analyzed the differences in TME between 44 normal subjects and 502 HNSCC patients. Finally, these six genes were further validated by samples in GEO database and our patient tissues *via* IHC staining. TCGA and GEO databases with large sample sizes provided us with adequate samples and comprehensive clinical data for signature establishment and validation, which significantly improved the accuracy and reliability of the prognostic model (17). Finally, we verified the prognostic value of this HRG-based signature utilizing patient samples from our hospital, which made the results more compelling and reliable. Overall, the expression levels of these HRGs can help evaluate the prognosis of HNSCC patients.

Of the six HRGs, three HRGs (STC1, HK1, and PGK1) have been reported to be related to the formation and progression of HNSCC. A study showed that STC1 and HK1 were both glycolysis-related genes with significant prognostic value in HNSCC (40). Zhang Y et al. demonstrated that hypoxia can increase the abundance of PGK1, which increased glycolysis and EMT by activating AKT signaling in OSCC (41). Cytoplasmic CDKN1B can be a potential biomarker for predicting prognosis and developing targeted therapeutic approaches (42). HS3ST1 was related to the NF-κB signaling pathway and selected to construct a prognostic signature for bladder cancer (43). In colorectal cancer, SRPX was used for the establishment of the prognostic model (44). These results suggested that our prognostic signature constructed by these six HRGs and the clinical characteristics can serve as a promising tool for HNSCC. Moreover, the additional validation in protein levels can largely increase the prediction value of this model. The spatial and temporal alterations of mRNAs, the local availability for protein biosynthesis, and post-transcriptional modification exert a strong influence on the correlation between protein and their encoding RNA levels (45). A recent study characterized the correlation between proteome and transcriptome and illustrated that phenotypes of genetic diseases can be explained by proteomic information, not transcript data. Positive correlations between protein and RNA levels were found in about half of the genes, and negative correlations were shown in 60 genes (46). IHC assay is an excellent method to validate signatures since pathologists can detect gene expression at the protein level within the context of tissue sections (47). IHC was also performed by other researchers to validate the expression of hub genes. For example, IHC data for HNSCC tissues and normal tissues from the HPA database were used to validate the differences in key genes expression (48, 49). The prognostic value of a ferroptosis-related signature was validated by IHC analysis (50).

There is an established correlation between hypoxia features and the TIME. It was reported that hypoxia promoted tumor immune escape and resistance (51). HIF signaling facilitates the recruitment and maintenance of pro-tumor immune cells and the inhibition of anti-tumor immune components, resulting in immune evasion (52). In general, the TME of HNSCC is largely infiltrated by immune cells, which mediates immune surveillance or escape by various molecular mechanisms (3). Moreover, multiple anti-cancer immune cells showed lower abundance in the high-risk group, such as B cells in Timer, MCP-count, EPIC, quanTIseq, and xCell algorithms. It was reported that B cells within TME were responsible for the production of antibodies and the promotion of T cell responses, including T cell priming, proliferation, and memory formation (53). Therefore, a higher abundance of B cells in the low-risk group can efficiently enhance the strength of anti-tumor immune responses and then inhibit tumor growth. These results confirmed the conclusions of previous studies. For instance, a recent study revealed that B cells showed a significantly high abundance in the low-risk group in HNSCC (54). Notably, a few types of immune cells showed a higher amount in the high-risk group, such as neutrophils in CIBERSORT, MCP-count, quanTIseq, and xCell algorithms. Proinflammatory neutrophils recruit monocytes and T regulatory cells *via* secreting chemokines such as CCL2 and CCL17. Consequently, T regulatory cells suppress other inflammatory T cell subpopulations, promoting tumor growth (55). Additionally, we evaluated ICI therapy efficacy. For example, the TIDE score is closely associated with ICI therapy responses. In our study, the Tide score and Tide Dysfunction score were elevated in the high-risk group, and this predicted a greater chance for immune evasion and ICI treatment resistance. These results suggested that treatment should be tailored according to patients’ comprehensive scores.

While some observations differ from the pioneering results. For instance, activated NK cells showed a higher abundance in the high-risk group, which probably came down that the hypoxic TME can impair the cell-killing function of NK cells (56). Mast cells are involved in various pathological processes under hypoxic conditions. For example, exposure of mast cells to ionomycin and substance P resulted in a significant initiation of the HIF1α signaling pathway (57). Therefore, the six-HRGs prognostic signature might mirror an ever-changing TIME for HNSCC patients in the high-risk group. To sum up, the above findings can at least partially shed a light on the underlying mechanisms concerning the worse outcomes in the high-risk group.

To demonstrate the advantages of our six-HRGs prognostic model over many existing ones, herein, we compared this signature with three published hypoxia signatures (22, 58, 59). As we can see, there is a lack of ROC curves to verify the predictive sensitivity and accuracy of the model (58). The contents of TME were poorly described and there was no external validation, such as data from the GEO database and clinical samples (22). Only CIBERSORT was used to describe the tumor microenvironment (59). By contrast, the present study provided a detailed description of TME and an analysis of the expression of immune checkpoint molecules. And more importantly, the data in the GEO database and clinical specimens were used as external validation sets to verify the robustness and accuracy of the model.

In summary, an HRG-based prognostic model with excellent capacity in assessing the overall outcomes of HNSCC patients was established in this study, which can assist clinicians to develop wise and comprehensive treatment strategies for HNSCC patients. Firstly, we established and verified a robust HRG-based prognostic model for HNSCC patients. Secondly, this hypoxia model not only provided a detailed evaluation of the TIME status of HNSCC patients in both high- and low-risk groups but determined potential ICI targets for the treatment of HNSCC. Thirdly, the expressions of hub HRGs were validated at both the protein level *via* IHC staining and the RNA level *via* databases. In addition to TCGA database, three GEO datasets and our patient samples were incorporated to further validate the prediction capacity of the established signature. The larger sample size allows for a more comprehensive assessment with minimal bias.

However, some limitations exist in the present study despite the advantages above mentioned. First, the HRG-based signature was constructed based solely on hypoxia-related hub genes, ignoring the critical fact that hypoxia TME as a cancer hallmark is associated with multiple gene networks. Therefore, the prediction value and clinical applicability of this HRG-based model are required to be further validated by combing it with other hallmarks, such as inflammation-related genes. Besides, there was no subgroup analysis for HNSCC samples in our study compared to recent studies (22) (59) and HR values of Cox regression analyses were not high (22). In addition, larger-size samples and multi-centric experimental researches are of great necessity to substantiate the prognostic value of hypoxia-related genes for HNSCC and biological functions.

## Conclusion

In conclusion, this work constructed a novel prognostic model based on six hypoxia-related genes in HNSCC patients. This prognostic signature was promising for the evaluation of immune cell infiltration and the efficiency of immune checkpoint therapy. Furthermore, the correlation of the HRG-based signature with survival status in multiple datasets and our samples revealed that it could be a powerful prognostic biomarker for HNSCC patients, and might be conducive to individualized management for HNSCC patients.

## Data availability statement

The raw data supporting the conclusions of this article will be made available by the authors, without undue reservation.

## Ethics statement

The studies involving human participants were reviewed and approved by The Ethics Committee of School and Hospital of Stomatology, Wuhan University. The patients/participants provided their written informed consent to participate in this study.

## Author contributions

S-RL designed the study, collected data, did statistical analysis, created the figures, and wrote the manuscript, Q-WM and BL performed results validation and revised the final manuscript. All authors contributed to the article and approved the submitted version.

## Funding

This work was supported by the National Natural Science Foundation of China (81872203 to BL); the National Natural Science Foundation of China (82001066 to Q-WM), and the Health Commission of Hubei Province scientific research project (WJ2021M175 to BL).

## Acknowledgments

We appreciate TCGA database for providing the original study data. We appreciate the contributions of patients to our study.

## Conflict of interest

The authors declare that the research was conducted in the absence of any commercial or financial relationships that could be construed as a potential conflict of interest.

## Publisher’s note

All claims expressed in this article are solely those of the authors and do not necessarily represent those of their affiliated organizations, or those of the publisher, the editors and the reviewers. Any product that may be evaluated in this article, or claim that may be made by its manufacturer, is not guaranteed or endorsed by the publisher.
