# Supplementary material for: Development and validation of a novel hypoxia-related signature for prognostic and immunogenic evaluation in head and neck squamous cell carcinoma
Source: Front Oncol. 2022 Nov 14;12:943945. doi: 10.3389/fonc.2022.943945 (PMC9702068; doi:10.3389/fonc.2022.943945)
Supplement: Supplementary file 1 [file DataSheet_1.docx]

**Supplementary Table 1. Demographic data of patients in the training cohort and validation cohort from TCGA database.**

|  |  |  | Training Cohort | | Validation Cohort | |
| --- | --- | --- | --- | --- | --- | --- |
|  |  |  | N | % | N | % |
| **Age (Years)** |  |  | 251 |  | 248 |  |
|  | ≥60 | 279 | 139 | 55.4 | 140 | 56.5 |
|  | <60 | 220 | 112 | 44.6 | 108 | 43.5 |
| **Gender** |  |  |  |  |  |  |
|  | Male | 366 | 185 | 73.7 | 181 | 73.0 |
|  | Female | 133 | 66 | 26.3 | 67 | 27.0 |
| **Grade** |  |  |  |  |  |  |
|  | 1 | 61 | 29 | 11.5 | 32 | 12.9 |
|  | 2 | 298 | 151 | 60.2 | 147 | 59.3 |
|  | 3 | 119 | 62 | 24.7 | 57 | 23.0 |
|  | 4 | 2 | 0 | 0 | 2 | 0.8 |
|  | X | 16 | 6 | 2.4 | 10 | 4.0 |
|  | Unknown | 3 | 3 | 1.2 | 0 | 0 |
| **Stage** |  |  |  |  |  |  |
|  | 1 | 25 | 14 | 5.6 | 11 | 4.5 |
|  | 2 | 79 | 39 | 15.5 | 40 | 16.1 |
|  | 3 | 89 | 50 | 19.9 | 39 | 15.7 |
|  | 4A | 287 | 136 | 54.2 | 151 | 60.9 |
|  | 4B | 13 | 6 | 2.4 | 7 | 2.8 |
|  | 4C | 3 | 3 | 1.2 | 0 | 0 |
|  | Unknown | 0 | 3 | 1.2 | 0 | 0 |
| **T** |  |  |  |  |  |  |
|  | 0 | 1 | 1 | 0.4 | 0 | 0 |
|  | 1 | 47 | 25 | 10.0 | 22 | 8.9 |
|  | 2 | 148 | 77 | 30.7 | 71 | 28.6 |
|  | 3 | 114 | 64 | 25.5 | 50 | 20.2 |
|  | 4 | 184 | 83 | 33.0 | 101 | 40.7 |
|  | X | 4 | 1 | 0.4 | 3 | 1.2 |
|  | Unknown | 1 | 0 | 0 | 1 | 0.4 |
| **M** |  |  |  |  |  |  |
|  | 0 | 484 | 245 | 97.6 | 239 | 96.4 |
|  | 1 | 4 | 3 | 1.2 | 1 | 0.4 |
|  | X | 11 | 3 | 1.2 | 8 | 3.2 |
| **N** |  |  |  |  |  |  |
|  | 0 | 212 | 107 | 42.6 | 105 | 42.4 |
|  | 1 | 75 | 42 | 16.7 | 33 | 13.3 |
|  | 2 | 197 | 97 | 38.7 | 100 | 40.3 |
|  | 3 | 9 | 3 | 1.2 | 6 | 2.4 |
|  | X | 5 | 2 | 0.8 | 3 | 1.2 |
|  | Unknown | 1 | 0 | 0 | 1 | 0.4 |
| **Race** |  |  |  |  |  |  |
|  | AI/AN | 2 | 2 | 0.8 | 0 | 0 |
|  | Asian | 10 | 3 | 1.2 | 7 | 2.8 |
|  | Black | 47 | 27 | 10.8 | 20 | 8.1 |
|  | White | 426 | 213 | 84.8 | 213 | 85.9 |
|  | Unknown | 14 | 6 | 2.4 | 8 | 3.2 |
| **HPV** |  |  |  |  |  |  |
|  | Unknown | 397 | 202 | 80.5 | 195 | 78.6 |
|  | Negative | 72 | 34 | 13.5 | 38 | 15.3 |
|  | Positive | 30 | 15 | 6.0 | 15 | 6.1 |
|  |  |  |  |  |  |  |
| **Smoke** |  |  |  |  |  |  |
|  | Yes | 378 | 192 | 76.5 | 186 | 75 |
|  | No | 111 | 54 | 21.5 | 57 | 23.0 |
|  | NA | 10 | 5 | 2.0 | 5 | 2.0 |
| **Sample Type** |  |  |  |  |  |  |
|  | Primary | 499 | 251 | 100 | 248 | 100 |
|  | Metastatic | 0 | 0 | 0 | 0 | 0 |

**Supplementary Table 2. Clinical characteristic of GEO database.**

| **GSE85446** | | **Number** |
| --- | --- | --- |
| gender | f | 27 |
|  | m | 39 |
| age | <60 | 33 |
|  | >=60 | 33 |
| Survival | Yes | 34 |
|  | No | 32 |
| pstagetnm | I | 6 |
|  | II | 14 |
|  | III | 10 |
|  | IV | 36 |
| pnstage | pos | 36 |
|  | neg | 30 |
| Recurrence | yes | 13 |
|  | no | 53 |

| **GSE41613** | | **Number** |
| --- | --- | --- |
| Sex | F | 31 |
|  | M | 66 |
| age | <60 | 50 |
|  | >=60 | 47 |
| tumor stage | I/II | 41 |
|  | III/IV | 56 |
| vital | Alive | 46 |
|  | Dead | 51 |
| fu time (year) | <3 | 40 |
|  | 3-5 | 19 |
|  | >5 | 38 |
| treatment | uni-modality | 43 |
|  | multi-modality | 53 |
|  | unknown | 1 |

| **GSE65858** | | **Number** |
| --- | --- | --- |
| gender | F | 47 |
|  | M | 223 |
| age | <60 | 153 |
|  | >=60 | 117 |
| smoking | Yes | 222 |
|  | No | 48 |
| alcohol | no | 31 |
|  | 1-30 | 84 |
|  | 31-60 | 63 |
|  | >60 | 92 |
| tumor_type | Primary | 253 |
|  | Secondary | 14 |
| tumor_site | Cavum Oris | 83 |
|  | Hypopharynx | 33 |
|  | Larynx | 48 |
|  | Oropharynx | 102 |
|  | NA | 4 |
| uicc_stage | I | 18 |
|  | II | 37 |
|  | III | 37 |
|  | IVA | 155 |
|  | IVB | 16 |
|  | IVC | 7 |
| t_category | 1 | 35 |
|  | 2 | 80 |
|  | 3 | 58 |
|  | 4a | 90 |
|  | 4b | 7 |
| n_category | 0 | 94 |
|  | 1 | 32 |
|  | 2a | 11 |
|  | 2b | 66 |
|  | 2c | 55 |
|  | 3 | 12 |
| distant_metastasis | 0 | 263 |
|  | 1 | 7 |
| treatment | mono | 78 |
|  | multi | 189 |
|  | palliative | 3 |

**Supplementary Table 3. Univariate Cox regression analysis.**

| **Gene** | **HR** | **95% CI** | ***P*-value** | |
| --- | --- | --- | --- | --- |
| ALDOA | 1.002 | 1.000-1.003 | 0.009 | |
| ANXA2 | 1.002 | 1.001−1.004 | 0.004 | |
| CDKN1B | 0.976 | 0.959−0.993 | 0.006 | |
| CXCR4 | 0.989 | 0.981−0.997 | 0.010 | |
| HK1 | 1.011 | 1.004−1.018 | 0.003 | |
| HS3ST1 | 1.096 | .026−1.170 | 0.007 | |
| HSPA5 | 1.002 | 1.001−1.004 | | 0.003 |
| LDHA | 1.002 | 1.000−1.003 | | 0.007 |
| LXN | 1.015 | 1.004−1.026) | | 0.006 |
| P4HA1 | 1.015 | 1.006−1.024 | | <0.001 |
| PCK1 | 1.181 | 1.066−1.308 | | 0.001 |
| PGK1 | 1.004 | 1.002−1.006 | | <0.001 |
| SRPX | 1.012 | 1.003−1.021 | | 0.007 |
| STC1 | 1.012 | 1.006−1.019 | | <0.001 |
| STC2 | 1.037 | 1.020−1.054 | | <0.001 |

**Supplementary Table 4. Coefficients of HRGs used for the prognostic model.**

| **Gene** | **Coefficient** |
| --- | --- |
| CDKN1B | -0.0419952346634971 |
| HK1 | 0.0158739494219758 |
| HS3ST1 | 0.0672361575623083 |
| PGK1 | 0.0025172232919511 |
| SRPX | 0.012653285301691 |
| STC1 | 0.01404817 |
